# Responses and relationship dynamics of men and their spouses during active surveillance for prostate cancer: health literacy as an inquiry framework

**DOI:** 10.1186/s12889-015-2068-8

**Published:** 2015-08-01

**Authors:** Lars Kayser, Nete S. Hansen-Nord, Richard H. Osborne, Anne Tjønneland, Rikke D. Hansen

**Affiliations:** The Danish Cancer Society Research Center, Strandboulevarden 49, DK-2100 Copenhagen Ø, Denmark; Department of Public Health, University of Copenhagen, Øster Farimagsgade 5, DK-1014 Copenhagen K, Denmark; Public Health Innovation, Population Health Strategic Research Centre, Deakin University, 221 Burwood Highway, Burwood Victoria, 3125 Melbourne Australia

**Keywords:** Health literacy, Prostate cancer, Spouses, HLQ

## Abstract

**Background:**

Early stage prostate cancer patients may be allocated to active surveillance, where the condition is observed over time with no intervention. Living with a cancer diagnosis may impose stress on both the men and their spouses.

In this study we explore whether the scores of and verbal responses to a Health Literacy Questionnaire can be used to identify individuals in need of information and support and to reveal differences in perception and understanding in health related situations within couples.

**Methods:**

We used the nine-domain Health Literacy Questionnaire (HLQ) as a framework to explore health literacy in eight couples where the men were on active surveillance for prostate cancer progression. Scores were calculated for each domain for both individuals. For each couple differences in scores were also calculated and related to the informants’ self-reported experiences and reflections in relation to participating in an active surveillance program. Also an inductive analysis was performed to identify themes in the responses and these themes were compared to those of HLQ.

**Results:**

The men tended to score higher than their spouses. There was no consistent relation between scores and the reported experiences and reflections.

However, some interesting patterns emerged, e.g. in two of the three couples with the largest within couple differences in HLQ scores, responses revealed discrepancies in how the men and their spouses perceived their situation.

Also, three themes emerged which related to six of the HLQ domains, i.e. involvement of spouses and other people around the men; support from and interaction with healthcare professionals; and use of the Internet for information retrieval.

**Conclusions:**

Using the HLQ as an interview framework provided insight into the differences within couples and provided new perspectives on their experiences, including their contact with health professionals and the patient-spouse interaction when dealing with prostate cancer. The HLQ used as a dialogue tool may be an adjunct to assist healthcare providers to understand the need for support and information of men with prostate cancer on active surveillance and the dynamics within couples.

## Background

When men are diagnosed with early stage prostate cancer (PCa) they may be allocated to active surveillance where the condition is observed over time with no intervention [1]. Living with a potentially harmful condition may lead to co-morbidities such as anxiety and depression [2, 3] and requires the ability to understand the situation and to actively engage with health professionals. In other words, these men need to be health literate [4]. The PCa diagnosis not only impacts the affected individual, but also his spouse. Partners of cancer patients are often the first to respond to the demands related to their partner’s illness [5–7]. Spouses of PCa patients on active surveillance may even have higher levels of anxiety than the patients themselves [8], potentially due to the lack of involvement or access to information or due to differences in how the men and their partners perceive and understand the situation.

Measuring health literacy may be used to assess PCa patients’ capability and resources to handle living with a cancer diagnosis. At the same time, health literacy assessment may permit the exploration of issues such as level of involvement, level of information or differences in perception and understanding of the cancer diagnosis between the men and their spouses. This paper introduces the concept of health literacy and a multidimensional questionnaire to measure health literacy - the Health Literacy Questionnaire (HLQ), a tool that brings new insight into the health situation of men with PCa and their spouses.

Health literacy has been defined as “*the cognitive and social skills which determine the motivation and ability of individuals to gain access to, understand and use information in ways which promote and maintain good health”* [9]. To date, a substantial body of evidence has emerged to demonstrate that an individual’s level of health literacy is associated with health-related outcomes [10]. Much of this research has focused on narrow elements of health literacy such as reading, numeracy and the ability to comply with and adhere to health advice and prescriptions. Based on these elements, low scores of health literacy have been associated with lower life expectancy and increased mortality rates in chronic conditions [10]. Such results have been informed by health literacy tests, including simple functional measures that provide information on an individual’s ability to read and calculate, such as the Test of Functional Health Literacy in Adults (TOFHLA) [11], Newest Vital Signs (NVS) [12] and Rapid Estimate of Adult Literacy in Medicine (REALM) [13]. However, health literacy is more than an individual’s performance in functional tests. Health literacy is also about the individual’s interaction with healthcare providers and the providers’ obligations to the patient [14]. It may even include the lifelong process of how individuals maintain good health and quality of life through engagement in health promotion, disease prevention and treatment [15]. Individuals who are unable to understand, access and use health information and health services, an indication of low health literacy, may be unable to achieve health- and treatment-related goals even if they are supported by health professionals [16, 17].

In 2013, Osborne et al. [18] introduced a new multidimensional tool to measure health literacy, the Health Literacy Questionnaire (HLQ). Its development was informed by the World Health Organization [9] and focuses on a wide range of health literacy abilities as well as the lived experience of engaging with practitioners and the healthcare system. It was constructed through extensive consultation with patients, practitioners and policymakers using a grounded approach including concept mapping [18]. It covers nine distinct domains to generate a full understanding of individuals’ self-reported skills, abilities, motivation and experiences in relation to their health and interaction with healthcare providers (Table 1).

**Table 1:** Description of the nine domains of the Health Literacy Questionnaire (HLQ)

Domain	Low level	High level
I: Feeling understood and supported by healthcare providers	People who are low on this domain are unable to engage with doctors and other healthcare providers. They don’t have a regular healthcare provider and/or have difficulty trusting healthcare providers as a source of information and/or advice.	Has an established relationship with at least one healthcare provider who knows them well and who they trust to provide useful advice and information and to assist them to understand information and make decisions about their health.
II: Having sufficient information to manage my health	Feels that there are many gaps in their knowledge and that they don't have the information they need to live with and manage their health concerns.	Feels confident that they have all the information that they need to live with and manage their condition and to make decisions.
III: Actively managing my health	People with low levels don’t see their health as their responsibility, they are not engaged in their healthcare and regard healthcare as something that is done to them.	Recognise the importance and are able to take responsibility for their own health. They proactively engage in their own care and make their own decisions about their health. They make health a priority.
IV: Social support for health	Completely alone and unsupported for health.	Their social system provides them with all the support they want or need for health.
V: Appraisal of health information	No matter how hard they try, they cannot understand most health information and get confused when there is conflicting information.	Able to identify good information and reliable sources of information. They can resolve conflicting information by themselves or with help from others.
VI: Ability to actively engage healthcare providers	Are passive in their approach to healthcare, inactive i.e., they do not proactively seek or clarify information and advice and/or service options. They accept information without question. Unable to ask questions to get information or to clarify what they do not understand. They accept what is offered without seeking to ensure that it meets their needs. Feel unable to share concerns. The do not have a sense of agency in interactions with providers.	Are proactive about their health and feel in control in relationships with healthcare providers. Are able to seek advice from additional healthcare providers when necessary. They keep going until they get what they want. Empowered.
VII: Navigating the healthcare system	Unable to advocate on their own behalf and unable to find someone who can help them use the healthcare system to address their health needs. Do not look beyond obvious resources and have a limited understanding of what is available and what they are entitled to.	Able to find out about services and supports so they get all their needs met. Able to advocate on their own behalf at the system and service level.
VIII: Ability to find good health information	Cannot access health information when required. Is dependent on others to offer information.	Is an ‘information explorer’. Actively uses a diverse range of sources to find information and is up to date.
IX: Understand health information well enough to know what to do	Has problems understanding any written health information or instructions about treatments or medications. Unable to read or write well enough to complete medical forms.	Can understand all written information (including numerical information) in relation to their health and able to write appropriately on forms where required.

The introduction of the HLQ enables us to investigate possible relations between health literacy and the capabilities and resources of couples, where men are enrolled in an active surveillance programme for early stage prostate cancer. The overall objective of the study was to explore whether the scores of and responses to a Health Literacy Questionnaire can be used to identify individuals in need of information and support and to reveal differences in perception and understanding in health related situations within couples. Furthermore, we sought to explore whether the health literacy domains constituting the HLQ emerged as themes important to the men and their spouses.

## Methods

The study uses a quantitative and a qualitative approach based on the nine domains of the HLQ. The scores of the HLQ are used to describe the level of the men and compare their level with that of their spouses. The qualitative part is based on the informants’ experiences and reflections, which they were instructed to share if an item evoked such responses. The theoretical framework of the analysis is based on WHO’s concept of health literacy [9] with the extended understanding inherent in the HLQ (Table 1) [18]. The qualitative analysis is inspired by thematic analysis [19].

### Study population

This investigation is a sub-study of the Danish feasibility study “Nordic Lifestyle Intervention Study among Men with Prostate Cancer” (NILS). NILS is a multicentre controlled trial initiated and led by authors RDH and AT from the Danish Cancer Society Research Center that covers aspects of behavioural lifestyle interventions, including vigorous exercise and a health-promoting diet among men with early stage PCa. The study is described in detail elsewhere [20, 21]. In brief, 24 Danish men with early stage PCa were enrolled and randomly assigned to an intervention group (n = 16) or a control group (n = 8) when they were diagnosed with early stage PCa or in connection with one of the regular meetings for clinical/biochemical monitoring during active surveillance for prostate cancer progression. The intervention included a minimum of three times 45 min of vigorous exercise per week and a health-promoting diet rich in whole grain rye (a minimum of 170 grams per day). The intervention phase was six months and the follow-up ended 12 months after enrolment. The participants were monitored individually four times during the NILS study through laboratory assessments at the clinical meetings in the active surveillance programme and through interviews and tests at meetings with a sports physiologist and a dietician, respectively. In addition, three informal evening get-togethers were held for the intervention group participants to give them the opportunity to meet each other and to inform them about the contents of the intervention. The participants’ spouses were encouraged to attend all of the meetings and the evening get-togethers.

The 14 participants of the intervention group who had completed the six-month intervention phase were invited by e-mail together with their spouses to participate in qualitative interviews. Eight of the 14 men (57 %) agreed to participate together with their spouses. The interviews were conducted in December 2012, which was three to nine months after end of the follow-up phase of the NILS study.

The demographic characteristics of the participants are presented in Table 2. The participants had been in a relationship with their partner for 20 to 46 years. Seven of the eight spouses joined all meetings during the NILS study together with their husband, and one spouse joined the clinical meetings and the evening get-togethers only.

**Table 2:** Demographic characteristics of the study participants, a subgroup of the NILS feasibility study

	Men	Spouses
Number of participants	8	8
Age (years)	55-70	55-68
Marital status		
- Married/In a relationship	8	8
Education^a^		
- Vocational training	1	5
- Short-cycle higher education	1	-
- Medium-cycle higher education	5	-
- Long-cycle higher education	1	3
Working status		
- Retired	2	3
- Employee	4	5
- Independent	2	-

^a^Duration of higher education: Short-cycle higher education: less than 2½ years. Medium-cycle higher education: less than 4 years. Long-cycle higher education: More than 4 years

### Health literacy questionnaire

The HLQ is a widely applied multidimensional measure of health literacy with nine domains, each contains four to six items with a total of 44 items. See Table 1 for a description of high and low scores for each domain. It was designed using a validity-driven approach and was validated in diverse samples of individuals in the community [18]. Response options in the first five domains have assigned values of 1 to 4 (agree/disagree scale) while domains six to nine have values of 1 to 5 (difficulty scales). The score for each domain is given as the average of the items belonging to the domain, with a high score indicating greater health literacy. A low score means that the informant has difficulties within the domain. The Danish adaptation of the HLQ was found to have high construct validity, homogeneity and reliability (personal communication, Terkildsen HM, 2014).

### Administration of the HLQ

All informants were interviewed by a researcher (NSH), who has been trained in qualitative methods, including interviews, during her five-year Master’s programme in Public Health. NSH was neither part of nor identified to be part of the clinical intervention, which should reduce the risk of the informants feeling a need to try to please the investigators.

All eight men and their spouses consented to take part in an interview, which was divided into three parts. The first two parts involved interviewing the men and their spouses separately using the HLQ as an interview framework. The third part was to interview the spouses about their roles during the NILS intervention. Results from the third part of the interview are reported elsewhere [21].

Only two persons, the interviewer and the informant, were present in the same room during each interview. In one case the spouse could hear the interview with the man and commented on a few items but did not influence the scoring. All sessions were digitally recorded for later transcription. For seven couples, the interviews took place in their homes, whereas one couple was interviewed at the Danish Cancer Society counselling centre, located close to their home, because they wished to be interviewed outside their home.

At the start of the interview, the participants were introduced to the aim of the study, and asked to respond to each item according to the scales and also to share experiences and reflections, if the item evoked any. If the informant hesitated while responding to an item, they were also asked whether the item precipitated any thoughts. The approach was similar to a cognitive testing [22] procedure, except that the focus was on the informant’s response to items and not on the understanding of the items. The informants were informed that their comments would remain anonymous and only be identifiable by the study authors for research purposes.

The interviewer went through the HLQ items with the informants according to the standard random order of the questionnaire. The informants filled in the questionnaire while reading it aloud. No one appeared to be dyslectic, but if they had been, the interviewer would have read it to them. If the informants were in doubt about how to respond or about the meaning of the questions the interviewer briefly advised the informant, ensuring a score for each item. The main comments were about similarities of the questions and the main questions were about what was meant by words such as “health professionals”. If the informant commented in relation to experiences or reflected on an item, the interviewer supported this as an open dialogue in order to ensure that the comments were properly documented on the recording. As the underlying domains are presented several times and in random order, the informants may come back to a certain theme several times during the interview. The duration of each interview varied from 35 to 70 min. The participants were not financially compensated for participating in the interviews.

### Data analysis

Each informant’s HLQ scores were calculated for each domain and an average for each domain was also calculated. Differences in scores within each couple were also calculated. The results were calculated by an assistant and anonymised to the researchers to ensure that they were not recognisable during the qualitative analysis.

The transcripts of the recorded interviews were analysed by the first author (LK). Each interview was read several times in alphabetical order to become familiar with each informant’s response. Then key concepts and preliminary themes for each informant were identified. Then the relation between the men and their spouses was revealed and the transcripts were read again pairwise to create a summary for each couple describing how the informants perceive their situation and relations.

A particular attention was paid to whether the responses revealed the informants’ perception of the nature of their relationship to their partners and a concordance between their experiences and reflections, as the dynamics within the couples was a particular interest of this study. The summaries were also used in an inductive process to identify themes and confirm those that emerged from the transcripts. The themes were merged into three overarching themes.

The content of the summaries from the couples were then compared to the HLQ scales according to inter- and intra-couple scores to explore relations between the reported experiences by the informants and the actual scores and differences within couples.

The final results were discussed with the interviewer (NSH) and the principle investigator (RDH). Descriptive data were presented for each individual, differences within couples and the mean values for each domain with ranges.

### Ethical considerations

The NILS study was approved by the Regional Ethical Committees on Human Studies in Copenhagen and Aarhus (H-1-2010-073) and by the Danish Data Protection Agency (2010-41-5520). This study was an add-on to the approved protocol. As the study does not involve biological material and is not related to other NILS data, no further approvals are needed.

## Results

The mean scores of all participants for the first four domains were 3.3 and 4.0-4.2 for domains six to nine (Table 3). Domain V: ‘Appraisal of health information’ recorded the lowest score of 3.0.

**Table 3:** HLQ scores in men with prostate cancer and in their spouses. Scores are given as a mean and with ranges

Domain	Men			Spouses			Both		
	Mean	Max.	Min.	Mean	Max.	Min.	Mean	Max.	Min.
I: Feeling understood and supported by healthcare providers	3.6	4.0	3.0	3.1	3.8	2.3	3.3	4.0	2.3
II: Having sufficient information to manage my health	3.4	4.0	3.0	3.3	4.0	2.5	3.3	4.0	2.5
III: Actively managing my health	3.5	4.0	2.4	3.1	4.0	2.2	3.3	4.0	2.2
IV: Social support for health	3.4	4.0	3.0	3.2	3.8	2.8	3.3	4.0	2.8
V: Appraisal of health information	2.9	3.6	2.2	3.1	3.8	2.6	3.0	3.8	2.2
VI: Ability to actively engage with healthcare providers	4.1	5.0	3.2	4.0	4.8	3.2	4.0	5.0	3.2
VII: Navigating the healthcare system	4.2	5.0	3.5	4.1	4.5	3.5	4.1	5.0	3.5
VIII: Ability to find good health information	4.2	5.0	3.8	4.2	4.6	3.8	4.2	5.0	3.8
IX: Understand health information well enough to know what to do	4.1	5.0	3.0	3.9	4.2	3.6	4.0	5.0	3.0

Overall, men tended to score higher than women. The largest difference was observed in domains I: ‘Feeling understood and supported by healthcare providers’ (0.5 unit difference) and III: ‘Actively managing my health’ (0.4 unit difference). For the remaining domains, men tended to score slightly higher or the same (0.0 to 0.2 unit difference), except for domain V: ‘Appraisal of health information’, where men scored 0.2 units lower than women.

Some patterns emerged when participants were grouped into couples. Overall, the men scored a little higher than their spouses and the differences in scores within couples were lower than the variations between couples (Table 4). Three couples stood out: both the husband and spouse of couples B and D scored lower than the other participants in most domains; and the spouse of couple G scored higher than her husband in eight of the nine domains.

**Table 4:** HLQ domain scores for the nine domains by couple

		Domain								
Couple		I: Feeling understood and supported by healthcare providers	II: Having sufficient information to manage my health	III: Actively managing my health	IV: Social support for health	V: Appraisal of health information	VI: Ability to actively engage with healthcare providers	VII: Navigating the healthcare system	VIII: Ability to find good health information	IX: Understand health information well enough to knpw what to do
A	Man	4.0	4.0	3.4	3.8	3.4	4.4	4.5	4.6	4.4
	Spouse	3.0	3.5	3.2	3.0	3.0	4.4	4.2	4.0	4.0
	Diff.	1.0	0.5	0.2	0.8	0.4	0.0	0.3	0.6	0.4
B	Man	3.0	3.0	2.4	3.0	2.6	3.4	3.5	3.8	3.2
	Spouse	2.3	2.5	2.2	3.4	2.6	3.2	3.5	3.8	3.8
	Diff.	0.7	0.5	0.2	−0.4	0.0	0.2	0.0	0.0	−0.6
C	Man	4.0	4.0	4.0	4.0	2.4	4.8	4.3	4.4	4.8
	Spouse	3.5	4.0	4.0	3.8	3.8	4.8	4.5	4.4	4.2
	Diff.	0.5	0.0	0.0	0.2	−1.4	0.0	−0.2	0.0	0.6
D	Man	3.3	3.3	3.2	3.2	2.6	3.2	4.0	4.2	3.0
	Spouse	2.8	3.0	2.6	3.0	2.8	3.6	4.0	4.0	3.8
	Diff.	0.5	0.3	0.6	0.2	−0.2	−0.4	0.0	0.2	−0.8
E	Man	3.8	3.5	4.0	3.4	3.6	5.0	5.0	5.0	5.0
	Spouse	3.8	3.8	3.2	2.8	3.2	3.6	4.0	4.6	4.2
	Diff.	0.0	−0.3	0.8	0.6	0.4	1.4	1.0	0.4	0.8
F	Man	3.8	3.0	3.6	3.2	2.2	4.0	4.0	4.0	4.4
	Spouse	3.0	3.3	3.0	3.0	3.0	4.0	4.0	4.0	3.6
	Diff.	0.8	−0.3	0.6	0.2	−0.8	0.0	0.0	0.0	0.8
G	Man	3.3	3.0	3.2	3.0	2.6	4.0	4.0	4.0	4.0
	Spouse	3.8	3.3	3.8	3.4	3.4	4.2	4.5	4.4	3.8
	Diff.	−0.5	−0.3	−0.6	−0.4	−0.8	−0.2	−0.5	−0.4	0.2
H	Man	3.8	3.5	4.0	3.6	3.6	4.0	4.0	3.8	4.0
	Spouse	2.8	2.8	3.0	3.0	2.6	3.8	4.0	4.0	3.8
	Diff.	1.0	0.8	1.0	0.6	1.0	0.2	0.0	−0.2	0.2

From the men’s responses, the following three main themes emerged:

1. Involvement of their spouses and people around them (related to domain IV: ‘Social support for health’).
2. Their support from and interaction with healthcare professionals (related to domains I: ‘Feeling understood and supported by healthcare providers’ and VI: ‘Ability to actively engage with healthcare providers’).
3. Their use of the Internet for information retrieval (related to domains VIII: ‘Ability to find good health information’, IX: ‘Understand health information well enough to know what to do’ and II: ‘Having sufficient information to manage my health’).

The above themes may indicate which focal reflections the informants had during the interview. Hence, these three themes are further explored as follows:

Theme 1 - Involvement of spouses and other people around the men

Most of the men expressed a wish to be discrete about their health condition and not to involve spouses, other relatives or friends in their problems. In spite of the intention to handle the situation by themselves, all men reported to various degrees that their spouses did accompany them to visits with healthcare professionals or were involved to some extent. Men A, C and F explicitly said that they relied on their spouses, and man G reported that he depended on his spouse in many situations.

Theme 2 - Support from and interaction with healthcare professionals

Men D and H distinguished themselves by reporting that they did not consult their general practitioner (GP). In contrast, man G explicitly indicated reliance on his GP. Spouse D, like her partner, also expressed extensive communication problems with her GP. The doctors and other health professionals involved in the NILS project were mentioned frequently and seemed to constitute a major part of the men’s health care. This was more pronounced for the men than their spouses.

Theme 3 - Use of the Internet for information retrieval

The men often used the Internet for PCa information retrieval except for man F, who relied on his spouse to get such information. The spouses were generally familiar with seeking information and using the Internet. However, their searching behaviour was not restricted to the PCa theme. Spouse H did not use the Internet much but found it easy to use.

### Relationship between HLQ scores and interviews

Overall, the scores within the domains did not relate strongly to the informants’ responses. In the cases of couples A, C, F and G, the men expressed that they relied on their spouses. While the spouses of couples C and G had higher scores than the men, supporting the two couples’ dynamic, this was not reflected in couples A and F.

However, two interesting patterns emerged. Two spouses (D and H) with the combination of an overall low HLQ score and a low score in domain I: ‘Feeling understood and supported by healthcare providers’ but not in domain VI: ‘Ability to actively engage with healthcare providers’ reported problems in their communication with their GP. In two of the three couples with the largest within couple differences (E and H), comments by the men or their spouses revealed discrepancies in how they perceived their situation.

## Discussion

The level of health literacy may influence health-related outcomes of men with PCa and health literacy has been shown to be strongly correlated with a wide range of self-management and health outcomes [10]. As health literacy is a multi-dimensional concept covering the ability not only to read and write but also to understand, process and appraise health information and interact with health professionals and others [15, 18], it is therefore a reasonable hypothesis that measurements of health literacy can help healthcare providers understand the needs and capabilities of the individuals they are in contact with.

This study was a first attempt to explore whether the HLQ with its nine distinct domains could be used as a tool to identify individuals who may be in need of support or information in order to be able to manage their own health conditions. We chose to start our exploration in a group of eight men with an early stage of PCa on an active surveillance programme [20] and enrolled in a behavioural lifestyle intervention, which included thorough information about the PCa prognosis. Given the intervention, this particular group of participants and their spouses would be expected to have a high level of access to resources and the capability to manage their situation, due to their active involvement in the six-months NILS intervention programme. This has been confirmed in the parallel study, in which the same study population appears to have overcome most of the challenges of living with PCa or being the partner of a PCa patient [21]. As expected, most of the participants had reasonably high scores across the HLQ scales, possibly due to the participation in the lifestyle intervention programme. This is supported by the results of the Danish HLQ validation study, in which a comparable group of middle-aged (50+ years) participants produced a lower health literacy score than that of this study (personal communication, Terkildsen HM, 2014).

The relatively higher scores among men than their spouses in domains I: ‘Feeling understood and supported by healthcare providers’ and III: ‘Actively managing my health’ may be explained by the men’s more direct involvement through the participation in the intervention study and the frequent meetings with healthcare professionals during the study. The higher scores among men may also be the result of their direct exposure to the challenge of having a disease rather than a gender related difference. Further studies in other settings, for instance, with women with breast or cervical cancer and their spouses, may shed more light on this issue. As expected, the informants’ reports of experiences and their reflections provided further insights into what can be achieved by the quantitative HLQ.

Most of the men tended not to share their health issues with others, including their spouses and relatives. This finding may be of importance to understand why spouses in other studies have been reported as having elevated anxiety [5] when the patients do not disclose all their thoughts and feelings. We were not able to retrieve this pattern through the health literacy scores in the relevant domain IV: ‘Social support for health’, which potentially should capture such issues with items such as “I have at least one person who can come to medical appointments with me” and “When I feel ill, the people around me really understand what I am going through”.

Although the men apparently preferred to be alone with their condition, the actual involvement of the spouses in the NILS programme, may not only give the spouses an increased insight but may also strengthen the men [7, 21].

The interaction with healthcare providers was another theme that related to both the GP and the healthcare professionals involved in the NILS study. It is of interest that the men particularly mentioned the health professionals of the NILS study and that two of them did not use their own GP. This may be explained by the relationship that has developed between the men and the health professionals in the course of NILS whereas the spouses were involved to a lesser degree and were not dependent on the health professionals, as they were not diagnosed with cancer. This illustrates how an area such as feeling support from health professionals may be related to whether an individual is directly involved as a patient or indirectly involved as a spouse.

It is interesting but not surprising that while the men mainly looked for information about their disease, most spouses reported using the Internet to seek information related to their own situation. The spouses were instructed to respond to the HLQ from a personal point of view and therefore their interest in their own health and not their partners’ could be a likely response to this framing.

Although it could be expected that the three themes that emerged from the verbal responses to the HLQ (involvement of spouses, support and interaction with healthcare professionals and information retrieval) relate to all the nine domains of the HLQ it should be noted that the themes only relate to six of the domains. In this way the themes summarize what seems to be of importance for the men in our study.

The importance of these areas may either be a result of their participation in the clinical program, NILS, or reflect their general concerns and needs from living with an early stage of PCa on active surveillance. The remaining three domains, III: 'Actively managing my health', V: 'Appraisal of health information' and VII: 'Navigating the healthcare system did not emerge'. A reason for this may be the affiliation with the clinical program, which may have resulted in a relation to the health professionals which reduces the self-directed behaviour and also facilitates the navigation of the healthcare system.

Based on our results we suggest that healthcare providers taking care of men with early stages of PCa collaborate with GPs and other healthcare providers to offer services for men and their spouses to communicate better with others and find information related to their condition. This will be in accordance with the recommendations for chronic care, for example, WHO’s model of Innovative Care for Chronic Conditions [23] and the proposed framework for managing patients with PCa [4].

Overall, the HLQ scores of this study could not be consistently related to the participants’ self-reported experiences or reflections during the interviews. Nonetheless, in some cases low scores related well to self-reported problems in the interaction with health professionals and also the differences in scores within a few couples related to their dynamics.

Furthermore, the spouses that expressed problems in the communication with their GPs were characterised by a low health literacy score, a lower score in domain I: ‘Feeling understood and supported by healthcare providers’ and an approximately average score in domain VI: ‘Ability to actively engage with healthcare providers’. This pattern may be an indicator of individuals having problems communicating with healthcare providers despite feeling in control of the consultation process. This pattern should be explored in larger studies as it may identify individuals that feel empowered but at the same time demonstrate low health literacy and therefore might be in need of support and understanding [16].

Since the HLQ was filled out together during a dialogue with a researcher the interaction between the interviewer and the participants may lead to variation in the depths of the participants’ verbal responses and may be considered a limitation of this study. Another limitation may be that the analysis of the interviews was performed by only one researcher. While the interpretation of the results was discussed with other authors and the coding was undertaken with blinding, independent analyses by additional researchers may have made the results more robust.

A strength is that despite of the relatively low number of participants and a convenience sampling we were able to recruit informants ranging from high to low health literacy and with both concordance and discordance between the couples in terms of the various domains of health literacy. However we are not able to document that we have reached saturation in relation to identify themes.

The study would have been strengthened by a larger number of couples and the inclusion of a wider range of health literacy profiles. Future studies would be improved by broader coverage and it will be necessary to extend our study to a larger population and it would be beneficial to include other conditions affecting women or a condition that is not gender related.

## Conclusions

The use of the HLQ as an interview framework may offer an insight into the differences within couples and provide new insight into their experiences, including their contact with health professionals and the patient-spouse interaction when dealing with an early stage of prostate cancer. The HLQ used as a dialogue tool may be an adjunct to assist healthcare providers to understand the need for support and information of men with PCa on active surveillance and the dynamics within couples.
